# Perception of stress and its association with socioeconomic, behavioral and clinical characteristics of woman, Vitória, 2022: a cross-sectional study

**DOI:** 10.1590/S2237-96222025v34e20240224.en

**Published:** 2025-08-04

**Authors:** Adriana Geraldina Vicente da Silva, Fernanda Garcia Gabira Miguez, Loys Lene da Costa Siqueira, Franciéle Marabotti Costa Leite

**Affiliations:** 1 Universidade Federal do Espírito Santo, Programa de Pós-Graduação em Saúde Coletiva, Vitória, ES, Brazil; 2 Universidade Federal do Espírito Santo, Departamento de Enfermagem, Vitória, ES, Brazil

**Keywords:** Women, Psychological stress, Socioeconomic factors, Analytical epidemiology, Cross-Sectional Studies, Mujeres, Estrés Psicológico, Factores Socioeconómicos, Epidemiología Analítica, Estudios Transversales

## Abstract

**Objective:**

To verify the perception of stress among women living in Vitória, Espírito Santo, and its association with socioeconomic, behavioral and clinical characteristics.

**Methods:**

Cross-sectional, population-based study with 1,086 women aged 18 or over, living in Vitória, Espírito Santo. Perceived stress levels were assessed using the Perceived Stress Scale tool and descriptive and multivariate analyses were performed using simple and multiple linear regression. Stata, version 17.0, was used.

**Results:**

The average perception of stress among women living in Vitória was 16.4 with standard deviation of 7.5. Women aged 60 or over presented, on average, 6.0 points less stress perception compared to younger women (18 to 24 years old). The group belonging to the first quartile of family income presented, on average, 1.4 point more stress, and those receiving government financial assistance showed 1.9 more point of stress. Women without religion had, on average, 1.2 more point of stress. Higher stress levels were found among those who did not practice physical activity, smokers and those with multimorbidity (p-value>0.050).

**Conclusion:**

There is a significant association between the perception of stress and socioeconomic, behavioral and clinical characteristics among women in Vitória.

## Resumen

**Objetivo:**

Verificar la percepción de estrés entre los residentes de Vitória, Espírito Santo, y su asociación con características socioeconómicas, comportamentales y clínicas.

**Métodos:**

Estudio transversal, basado en la población, con 1086 mujeres de 18 años o más, residentes en Vitória, Espírito Santo. Los niveles de estrés percibido se evaluaron mediante el instrumento Perceived Stress Scale y se realizaron análisis descriptivos y multivariables mediante regresión lineal simple y múltiple. Se utilizó Stata, versión 17.0.

**Resultados:**

La percepción media de estrés entre las mujeres residentes en Vitória fue de 16,4 y la desviación estándar de 7,5. Las mujeres de 60 años o más tenían, en promedio, 6,0 puntos menos de estrés percibido en comparación con las mujeres más jóvenes (18 a 24 años). El grupo perteneciente al primer cuartil de ingresos familiares tuvo, en promedio, 1,4 punto más de estrés, y quienes reciben asistencia del gobierno, 1,9 punto más de estrés. Las mujeres sin religión presentaron, en promedio, 1,2 puntosmás de estrés. Se encontraron mayores medias de estrés entre quienes no practicaban actividad física, fumadores y personas con multimorbilidad (p-valor>0,050).

**Conclusión:**

Existe una asociación significativa entre la percepción del estrés y las características socioeconómicas, comportamentales y clínicas de las mujeres de Vitória.

**Ethical aspects**

This research respected ethical principles, having obtained the following approval data::

Research Ethics Committee: Universidade Federal do Espírito Santo

Opinion number: 4.974.080

Approval date: 14/9/2021

Certificate of Submission for Ethical Appraisal: 88138618000005060

Informed Consent Form: Obtained from all participants prior to collection.

## Introduction

Stress theory suggests that stress arises when a situation is perceived as threatening and the individual believes that the demands imposed exceed their ability to cope (1). A meta-analysis revealed that the prevalence of stress in the general population during the COVID-19 pandemic was 35% (2). Stressful events are processed differently by men and women, with women tending to experience higher levels of perceived stress (3). A study carried out in Brazil revealed that during social isolation, 21.5% of the population presented severe/extreme stress, with being a female standing as one of the risk factors associated with these high levels of stress (4).

The physiological responses that allow adaptation to stressful life events involve the hypothalamic-pituitary-adrenal axis and the autonomic nervous system, in addition to their complex interactions with the metabolic system and the pro- and anti-inflammatory mechanisms of the immune system (5-6). However, exposure to multiple stressors, hyperstimulation and dysregulation of these interactions result in degradation of the body and brain, which can lead to changes in health conditions (5-7). Therefore, chronic stress can negatively affect both physical and mental health, contributing to disorders such as anxiety, depression, cardiovascular disease, obesity, metabolic syndrome and type 2 diabetes, as well as bone problems such as osteopenia and osteoporosis (8).

Stress is not just about what happens to a person, but about how that person perceives and reacts to what happens, being influenced by personal and contextual factors (9). A Brazilian study observed that socioeconomic factors, such as age between 18 and 20 years and being female, are associated with severe/extreme stress (4). Exposure to adverse socioeconomic conditions contributes to chronic stress, due to feedback loops that perpetuate difficulty in coping and the perception of stressors as uncontrollable. Therefore, addressing the socioeconomic context is essential for the effectiveness of psychological interventions (10-11).

Thus, some clinical factors, such as a history of anxiety, depression and medication use, are also associated with stress (4). A Canadian study demonstrated that individuals with cardiometabolic multimorbidity are more likely to report high levels of stress, with this being higher among those with three cardiometabolic conditions. Furthermore, there is an association between multimorbidity and lifestyle behaviors, such as physical activity and stress levels, with these individuals presenting lifestyle behaviors associated with negative health outcomes (12).

Given the above, this study aimed to assess the perception of stress among women living in Vitória, Espírito Santo, and its association with socioeconomic, behavioral and clinical characteristics.

## Methods

### Outline

This is a cross-sectional study, which is part of a larger study entitled “Violence against women: a population-based study in Vitória, Espírito Santo”.

### Context

The study was carried out in Vitória, in the state of Espírito Santo, Brazil. It has a population of 327.801 people, of which 53,04% are women (13).

### Participants

The study sample consisted of women, aged 18 or over, living in Vitória, who currently had or had an intimate partner in the last 24 months. 

For sample definition, the census sectors of the Brazilian Institute of Geography and Statistics of 2010 were selected. The selection involved dividing the total number of households (108,515) by 100, resulting in a systematic skip of 1,085. The initial sector was chosen randomly, and the other 99 subsequent sectors were based on systematic skipping. Households were randomly selected from a list from the Brazilian Institute of Geography and Statistics. In total, 1,086 women were interviewed, with one selected from each household.

### Variables

The independent variables analyzed were composed of socioeconomic data, such as: age group (years of age) (18 to 24; 25 to 34; 35 to 44; 45 to 59; 60 or older), race/skin color (white and non-white), education (years of study) (0 to 8 years; equal to or greater than 8 years), marital status (with partner; without partner), family income by quartile (1st quartile - poorest; 2nd quartile; 3rd quartile; 4th quartile - richest), home ownership (no; yes), religion (no; yes), paid work (no; yes), number of people in the household (0 to 2 people; 3 to 5 people; 6 or more people), receiving government financial assistance (no; yes) and health insurance (no; yes). Behavioral data are composed of: physical activity (no; yes), alcohol consumption (no; yes) and smoking (no; yes). Finally, the clinical data are: nutritional status (eutrophic, underweight, overweight, obesity) and multimorbidity (no; yes).

### Data source and measurement

The outcome of the present study assessed the levels of perceived stress using the Perceived Stress Scale tool, in its short form, which contains ten questions and allows the assessment of the perception of stress in relation to life experiences in the previous month (14). The scale used for this study was translated and validated in Brazil (15). The stress instrument used was the short version with ten questions, with response options ranging from 0 to 4 (0=never; 1=almost never; 2=sometimes; 3=almost always and 4=always) for questions with a negative connotation. Questions with a positive connotation have inverted scoring (0=4; 1=3; 2=2; 3=1 and 4=0). The scale total is the sum of the scores of these ten questions, and the score can vary from 0 to 40 (15).

### Data collection

The interviews were conducted at the women’s homes, from January to May 2022. In December 2021, a pilot study was conducted, interviewing 34 women who were not included in the final sample. Tablets devices managed with the help of the Research Electronic Data Capture electronic data capture tool were used for data collection.

### Statistical methods

The analyses were conducted by presenting raw and relative frequencies and 95% confidence intervals (95% CI). The presentation of the mean (µ) for continuous variables together with their standard deviation (SD) was used as a measure of central tendency. The normality of continuous data was verified by viewing the histogram and Skewness/Kurtosis confirming the normal distribution and non-rejection of the null hypothesis by the Shapiro-Francia test, equal to 0.14. The data coefficient was evaluated using Cronbach’s Alpha Scale (α), showing good internal consistency (0.80), with confirmation by McDonald’s omega (ω) of 0.80. Then, the data were evaluated using bivariate analysis, using t-tests for the mean when the independent variables were dichotomous and analysis of variance by ANOVA for the means, when the independent variables presented three or more categories. At this stage, a p-value of 5% was considered significant. To verify the proposed association, multivariate analyses by simple and multiple linear regression were expressed by their betas (ß). For this stage, the significance level to enter the multivariate model was 20% and 5% to remain in the final model. All steps described in the analysis were performed using the Stata statistical software, version 17.0. 

## Results

The mean (µ) of stress perception among women living in Vitória was 16.4 and the standard deviation (SD) was 7.5. The highest means (µ=2.0; SD 1.2) were found when women were asked about being nervous or stressed (µ=2.0; SD 1.2), and when asked about feeling angry because of things that were beyond their control (µ=2.0; SD 1.3) (Table 1).

**Table 1:** Mean, median and standard deviation (SD) of perceived stress among women. Vitória, 2022 (n=1,086)

In the last month, how often	D^a^	Mean±SD	Median
Have you been upset because of something that happened unexpectedly?	-	1.7±1.3	2.00
Have you felt like you were unable to control important things in your life?	-	1.5±1.3	1.00
Have you been nervous or stressed?	-	2.0±1.2	2.00
Have you been confident in your ability to deal with your personal problems?	+	1.1±1.1	1.00
Did you feel like things turned out the way you expected?	+	1.9±1.2	2.00
Did you think you couldn’t handle all the things you had to do?	-	1.8±1.2	2.00
Have you been able to control annoying issues in your life?	+	1.4±1.2	1.00
Did you feel like every aspect of your life was under control?	+	1.7±1.2	2.00
Have you been angry about things that were out of your control?	-	2.0±1.3	2.00
Have you felt like your problems have piled up so much that wouldn’t be able to solve them?	-	1.3±1.3	1.00
**PSS-10 total score**		16.4±7.5	17.00

^a^ D: Scale domains, with negative (-) being responses with negative connotations and positive (+) being responses with positive connotations; ^b^PSS-10: Perceived Stress Scale-10.

The highest average perception of stress was found among women aged 18 to 24 (µ=20.3), non-white (µ=16.8), with 8 years of schooling or more (µ=16.7), belonging to the first income quartile (µ=17.5), without partners (µ=17.8), non-religious (µ=16.0), who live with six or more people in the same household (µ=17.6), who do not have health insurance (µ=17.0), do not own a home (µ=17.5), receive government financial assistance (µ=18.1), do not do physical activity (µ=17.2), are smokers (µ=17.2) and drink alcohol regularly (µ=17.0) (Table 2).

**Table 2:** Raw frequency (n), relative frequency, mean and standard deviation (SD) of stress perception according to socioeconomic, behavioral and clinical characteristics of women. Vitória, 2022 (n=1,086)

Variables	n^a^ (%^b^ )	Mean±SD	p-value
**Age group** (years)			<0.001
18-24	105 (9.6)	20.3±7.0	
25-34	192 (17.6)	18.1±6.9	
35-44	227 (20.9)	17.4±7.9	
45-59	300 (27.7)	15.4±7.4	
60 (or older)	262 (24.1)	13.9±6.9	
**Race/skin color**			0.040
Not White	650 (40.2)	16.8±7.8	
White	436 (59.8)	15.8±7.3	
**Education** (years)			0.023
<8	203 (18.7)	15.3±7.6	
≥8	883 (81.3)	16.7±7.5	
**Household income** (quartile)			0.003
1st (poorest)	290 (26.7)	17.5±7.6	
2nd	288 (26.5)	16.2±7.3	
3rd	238 (21.9)	16.7±7.3	
4th (richest)	270 (24.9)	15.2±7.6	
**Marital status**			0.004
With partner	886 (81.6)	16.1±7.5	
Without a partner	200 (18.4)	17.8±7.3	
Religion			<0.001
No	182 (16.8)	18.5±8.3	
Yes	904 (83.2)	16.0±7.3	
**Paid work**			0.531
No	598 (55.1)	16.3±7.5	
Yes	488 (44.9)	16.7±7.8	
**Number of people at home**			0.004
0-2	412 (37.9)	15.5±7.5	
3-5	624 (57.5)	16.9±7.5	
≥6	50 (4.6)	17.6±6.2	
**Has a health plan**			0.011
No	514 (47.3)	17.0±7.7	
Yes	572 (52.7)	15.9±7.7	
**Owns own home**			0.002
No	317 (29.2)	17.5±7.9	
Yes	769 (70.8)	15.9±7.3	
**Government financial assistance**			<0.001
No	895 (82.4)	16.0±7.3	
Yes	191 (17.6)	18.1±8.2	
**Physical activity**			<0.001
No	589 (54.2)	17.2±7.6	
Yes	497 (45.8)	15.5±7.3	
Smoking			0.029
No	804 (74.0)	16.1±7.5	
Yes	282 (26.0)	17.2±7.5	
**Consumption of alcohol**			0.008
No	548 (50.7)	15.8±7.45	
Yes	532 (49.3)	17.0±7.6	
**Nutritional status**			0.078
Eutrophy	449 (41.4)	16.4±7.4	
Low weight	33 (3.0)	16.2±7.6	
Overweight	356 (32.8)	15.8±7.6	
Obesity	246 (22.8)	17.4±7.6	
Multimorbidity			0.196
No	598 (55.1)	16.1±7.2	
Yes	488 (44.9)	16.7±7.8	

The raw analysis of Table 3 shows that the perception of stress was related to age, education, family income, marital status, religion, number of people in the household, whether the individual has health insurance, owns a home and receives government financial assistance, as well as the practice of physical activity, smoking and the use of alcoholic beverages (p-value<0.05) (Table 3).

**Table 3:** Simple linear regression coefficient (β) and 95% confidence intervals (95%CI) of the association between stress perception and socioeconomic, behavioral, and clinical characteristics among women. Vitória, 2022 (n=1,086)

Variables	β (95%CI)	p-value
**Age group** (years)		<0.001
18-24	1.0	
25-34	-2.20 (-3.93; -0.47)	
35-44	-2.96 (-4.64; -1.28)	
45-59	-4.90 (-6.51; -3.28)	
60 (or older)	-6.42 (-8.07; -4.78)	
**Race/skin color**		0.040
Not White	0.95 (0.43; 1.86)	
White	1.0	
**Education** (years)		0.023
<8	1.33(0.19; 2.48)	
≥8	1.0	0.003
**Household income** (quartile)		
1st (poorest)	2.28 (1.04; 3.52)	
2nd	0.96 (-0.29; 2.20)	
3rd	1.52 (0.22; 2.82)	
4th (richest)	1.0	
**Marital status**		0.004
With partner	1.0	
Without a partner	1.68 (0.53; 2.82)	
**Religion**		<0.001
No	2.55 (1.36; 3.74)	
Yes	1.0	
**Paid work**		0.530
No	-2.87 (-1.19; 0.61)	
Yes	1.0	
**Number of people in the household**		0.004
0-2	1.0	
3-5	1.47 (0.54; 2.40)	
6 (or more people)	2.14 (-0.55; 4.34)	
**Has a health plan**		0.011
No	1.16 (0.26; 2.05)	
Yes	1.0	
**Owns own home**		0.002
No	1.54 (0.56; 2.52)	
Yes	1.0	
**Government financial assistance**		<0.001
No	1.0	
Yes	2.08 (0.91; 3.25)	
**Physical activity**		<0.001
No	1.65 (0.76; 2.54)	
Yes	1.0	
Smoking		0.029
No	1.0	
Yes	1.31 (0.11; 2.15)	
**Alcohol consumption** (n=1,080)		0.008
No	1.0	
Yes	1.22 (0.32-2.11)	
**Nutritional status** (n=1,084)		0.078
Eutrophy	1.0	
Low weight	-1.07 (-2.76; 2.55)	
Overweight	-0.55 (-1.60; 0.49)	
Obesity	1.06 (-0.10; 2.23)	
Multimorbidity		0.196
No	1.0	
Yes	0.59 (-0.30; 1.49)	

In the adjusted analysis, presented in Table 4, women aged 60 or over presented, on average, 6.04 points less stress perception, compared to younger women (18 to 24 years old). The group belonging to the first quartile of family income, that is, the poorest, presented, on average, 1.35 point more stress, when compared to the richest, and those who receive government financial assistance perceived 1.86 point more stress, compared to those who do not receive assistance. Women without religion had, on average, 1.21 more points of perceived stress compared to women who have a religion. Higher stress levels were found among women who did not practice physical activity (β=1.46), declared themselves to be smokers (β=1.22) and had some multimorbidity (β=2.38) (p-value<0.050) (Table 4).

**Table 4:** Multiple linear regression coefficient (β) and 95% confidence intervals (95%CI) of the association between stress perception and socioeconomic, behavioral and clinical characteristics among women. Vitória, 2022 (n=1,086)

Variables	Adjusted β (95%CI)	p-value
**Age group** (years)		<0.001
18-24	1.0	
25-34	-2.23 (-3.95; -0.51)	
35-44	-2.79 (-4.46; -1.11)	
45-59	-4.63 (-6.26; -3.00)	
60 (or more)	-6.04 (-7.71; -4.38)	
**Race/skin color**		0.799
Not White	0.12 (-0.83; -1.07)	
White	1.0	
**Education** (years)		0.280
<8	0.67 (-0.55; -1.89)	
≥8	1.0	
**Household income** (quartile)		0.003
1st (poorest)	1.35 (0.92; -2.61)	
2nd	0.96 (-0.25; -2.17)	
3rd	1.58 (0.32; -2.85)	
4th (richest)	1.0	
**Marital status**		0.142
With partner	-0.85 (-1.98; -0.28)	
Without a partner	1.0	
Religion		0.046
No	1.21 (0.19; -2.40)	
Yes	1.0	
**Number of people at home**		0.110
0-2	1.0	
3-5	1.02 (0.07; -1.97)	
6 (or more people)	0.48 (-1.70;-2.65)	
**Has a health plan**		0.805
No	-0.14 (-1.23; -0.95)	
Yes	1.0	
**Owns own home**		0.770
No	0.15 (-0.86; -1.16 )	
Yes	1.0	
**Government financial assistance**		0.001
No	1.0	
Yes	1.86 (0.73; -2.99)	
**Physical activity**		0.001
No	1.46 (0.60; -2.32)	
Yes	1.0	
Smoking		0.015
No	1.0	
Yes	1.22 (0.23; -2.19)	
**Alcohol consumption** (n=1,080)		0.935
No	1.0	
Yes	0.04 (-0.86; -0.95)	
**Nutritional status** (n=1,084)		0.407
Eutrophy	1.0	
Low weight	-1.23 (-3.75; -1.30)	
Overweight	-0.06 (-1.06; -0.95)	
Obesity	0.67 (-0.46; -1.81)	
Multimorbidity		<0.001
No	1.0	
Yes	2.38 (1.44;-3.32)	

## Discussion

This study verified the average perception of stress among women living in Vitória (µ=16.4; SD 7.51). Higher stress levels are observed among younger women (18 to 24 years old), who belong to the poorest group, who receive government financial assistance, and who declare that they have no religion. Furthermore, not practicing physical activity, being a smoker and having some multimorbidity were associated with increased stress among the participants.

Age was negatively associated with stress levels, with younger women (18-24 years) reporting the highest levels. A population-based study carried out in a municipality in southern Brazil revealed that women, especially in the 18 to 39 age group, constituted one of the most stressed groups (16). This phenomenon can be attributed to the accumulation of responsibilities in multiple social, family and professional roles, in addition to personal, biological and social demands, contributing significantly to increased stress (17). Similarly, a study with elderly people from São Paulo and Paraíba showed that in general they considered themselves more satisfied with life compared to younger people, suggesting that, according to the theory of socioemotional selectivity, emotions are better regulated with aging, resulting in a greater feeling of well-being (18).

Corroborating our results regarding family income, a Danish study that investigated the association between perceived stress, socioeconomic status and health risk behavior found that adjusted *odds ratios* (OR) of perceived stress were higher among residents with low monthly income (OR 1.57; 95%CI 1.35; 1.83), compared to those with high monthly income (19). This finding can be justified by the concept of financial strain, defined as a specific type of stress caused by economic difficulties, such as financial instability, accumulation of debts and impaired credit history (20). Although poverty is directly related to income, financial strain is a subjective emotional experience, unrelated to a fixed income amount (21).

Both poverty and financial stress negatively affect mental health, as previous studies have shown (22-23). In the case of mothers, financial stress is associated with an increase in symptoms of depression (24). In families with young children, this economic pressure can lead to difficult decisions, compromising the quality of parental care and increasing feelings of guilt, which further exacerbates mental health problems (25).

Another relevant factor is government support, as women who reported receiving government financial assistance reported higher levels of stress compared to those who did not receive such assistance. Thus, a study with 433 participants registered in the Federal Government’s Unified Registry in Belém, including mothers or guardians of children aged 5 to 18, indicated that 96.5% of the main caregivers were women. This study showed that poorer families had higher levels of parental stress, that is, the stress associated with the responsibilities and demands of caring for children. These data reinforce that families eligible for government financial assistance were more economically vulnerable, corroborating the previous discussion on the impacts of poverty on mental health (26).

In addition to income and social support, participants without a religion showed a greater perception of stress compared to those with religious beliefs. A study with 795 people investigated how spiritual connections helped in coping with stress and anxiety in the early stages of the COVID-19 pandemic. Women and young people are more vulnerable to stress and anxiety; however, spiritual connections can act as a protective resource, especially in times of crisis, reducing the likelihood of symptoms of stress and anxiety. Women’s greater susceptibility to stress may be linked to heightened reactivity and less effective use of coping strategies (27). Although spiritual connection offers comfort, it may serve more as a temporary relief than a lasting solution for this group (28).

Thus, spirituality and religiosity can be agents of relief in times of stress, acting as adaptation strategies that increase resilience throughout life and contribute to maintaining quality of life and health (29).

Regarding physical activity, women who do not practice physical activities presented a higher perception of stress than those who exercise regularly. A study carried out in Aracaju, with a predominantly female sample (51%), revealed that lack of physical activity is associated with higher levels of stress (30). Regular physical activity is associated with improvements in self-esteem, self-concept, perception of body image, cognitive functions and social relationships, in addition to contributing to the reduction of stress and anxiety. Incorporating healthy habits is essential to promoting mental health. Thus, physical activity emerges as a crucial tool for well-being (31).

Smoking is associated with greater perception of stress. Women who smoke or have smoked in the past reported, on average, 1.23 more point of stress compared to non-smokers, confirming the findings of previous studies (32-33). Smoking is often used as a coping mechanism for stress, providing a respite from adverse environments such as poor housing, unfavorable neighborhoods, or traffic situations (32). This practice is seen as a strategy to alleviate everyday issues (33).

Regarding health conditions, women diagnosed with multimorbidity reported a greater perception of stress compared to those without multiple diagnoses, which may be justified by the suffering related to diseases and daily stressors faced by people with chronic conditions (34-35). In a population-based cross-sectional study of older adults in low- and middle-income countries, it was highlighted that chronic conditions and multimorbidity were associated with higher levels of stress. Furthermore, perceived stress, combined with chronic conditions, has been shown to be related to worse health outcomes, highlighting the urgent need for integrated, low-cost population-level interventions to address stress in individuals with chronic diseases (36).

These findings highlight the complexity of stress. Studies that explore the perception of this health problem in relation to socioeconomic, behavioral and clinical characteristics offer a more detailed understanding of the causes and impacts of this phenomenon (26,37). Stress, “an invisible villain”, requires public policies that address social inequalities and promote effective coping strategies to mitigate its effects. Measures such as promoting gender equality, access to education, employment, healthy food, leisure and physical activities are fundamental. Furthermore, stress assessment by healthcare professionals is essential to identify comorbidities and enable early interventions. The inclusion of psychologists in family health teams can further expand access to mental health care (38).

The study has limitations that need to be considered, such as the cross-sectional design that makes it impossible to infer causality, only allowing the projection of possible associations between the data evaluated. However, it comes from a population-based survey representative of the population, strengthening the relationships examined. Another limitation is the possibility of information bias, which may influence the stress perception evaluation. However, the data collection methodology uses a validated instrument for screening and is similar to that of other studies on the subject, and the findings of the present study are in line with the literature.

Finally, this study allows us to conclude that the socioeconomic, behavioral and clinical profile of women is associated with the phenomenon of stress. Therefore, it is important to promote the development of more effective intervention strategies and public policies, which address not only the physical but also the psychological aspects of stress, considering the different cultural contexts and their specific demands. In this sense, this holistic perspective of women has the potential to transform the approach to this group, integrating it into a broader context and promoting advances capable of positively impacting women’s lives. It is important to consider that although challenging, this proposal also represents a unique opportunity to lead significant and sustainable changes in health care.

## Data Availability

The database and analysis codes used in the research are available at https://doi.org/10.48331/scielodata.C8WFB1.
