# Supplementary material for: Perception of stress and its association with socioeconomic, behavioral and clinical characteristics of woman, Vitória, 2022: a cross-sectional study
Source: Epidemiol Serv Saude. 2025 Aug 4;34:e20240224. doi: 10.1590/S2237-96222025v34e20240224.en (PMC12334160; doi:10.1590/S2237-96222025v34e20240224.en)
Supplement: Translation

# Translation: Percepção de estresse e sua associação com as características socioeconômicas, comportamentais e clínicas de mulheres, Vitória, 2022: estudo transversal

**DOI:** 10.1590/S2237-96222025v34e20240224.pt

**Authors:** Adriana Geraldina Vicente da Silva, Fernanda Garcia Gabira Miguez, Loys Lene da Costa Siqueira, Franciéle Marabotti Costa Leite

**Affiliations:** 1 Universidade Federal do Espírito Santo, Programa de Pós-Graduação em Saúde Coletiva, Vitória, ES, Brasil; 2 Universidade Federal do Espírito Santo, Departamento de Enfermagem, Vitória, ES, Brasil

**Keywords:** Mulheres, Estresse Psicológico, Fatores Socioeconômicos, Epidemiologia Analítica, Estudos Transversais

## Abstract

**Objetivo:**

Verificar a percepção de estresse entre as residentes de Vitória, Espírito Santo, e sua associação com características socioeconômicas, comportamentais e clínicas.

**Métodos:**

Estudo transversal, de base populacional, com 1.086 mulheres de 18 anos ou mais, moradoras de Vitória, Espírito Santo. Os níveis de estresse percebidos foram avaliados por meio do instrumento Perceived Stress Scale e foram realizadas análises descritivas e multivariáveis, por regressão linear simples e múltipla. Foi utilizado o programa Stata, versão 17.0.

**Resultados:**

A média de percepção de estresse entre as mulheres residentes de Vitória foi de 16,4 e desvio-padrão de 7,5. Mulheres com 60 anos ou mais apresentaram, em média, 6,0 pontos a menos de percepção de estresse em comparação às mais jovens (18 a 24 anos). O grupo pertencente ao primeiro quartil de renda familiar apresentou, em média, 1,4 ponto a mais de estresse, e aquelas que recebem auxílio do governo, 1,9 ponto a mais de estresse. Mulheres sem religião tiveram, em média, 1,2 ponto a mais de estresse. Maiores médias de estresse foram encontradas entre as que não praticavam atividade física, tabagistas e com multimorbidade (p-valor>0,050).

**Conclusão:**

Há uma associação significativa entre a percepção de estresse e características socioeconômicas, comportamentais e clínicas entre as mulheres de Vitória.

**Aspectos éticos**

A presente pesquisa respeitou os princípios éticos, obtendo os seguintes dados de aprovação: :

Comitê de ética em pesquisa: Universidade Federal do Espírito Santo

Número do parecer: 4.974.080

Data de aprovação: 14/9/2021

Certificado de apresentação de apreciação ética: 88138618000005060

Registro do consentimento livre e esclarecido: Obtido de todos os participantes antes da coleta.

## Introdução

A teoria do estresse aponta que ele surge quando os indivíduos percebem uma situação como ameaçadora e acreditam que as demandas impostas excedem sua capacidade de gerenciamento (1). Uma metanálise revelou que a prevalência de estresse na população geral, durante a pandemia de covid-19, foi de 35% (2). Os eventos estressantes são processados de maneira diferente por homens e mulheres, sendo que as mulheres tendem a experimentar níveis mais altos de estresse percebido (3). Um estudo realizado no Brasil indicou que, durante o isolamento social, 21,5% da população apresentou estresse severo/extremo, com o sexo feminino sendo um dos fatores de risco associados a esses níveis elevados de estresse (4).

As respostas fisiológicas que permitem a adaptação a eventos estressantes da vida envolvem o eixo hipotálamo-hipófise-adrenal e o sistema nervoso autônomo, além de suas interações complexas com o sistema metabólico e os mecanismos pró e anti-inflamatórios do sistema imunológico (5-6). No entanto, a exposição a inúmeros estressores, a hiperestimulação e a desregulação dessas interações resultam na degradação do corpo e do cérebro, o que pode ocasionar alterações na condição de saúde (5-7). O estresse crônico pode, portanto, afetar negativamente tanto a saúde física quanto a mental, contribuindo para transtornos, como ansiedade, depressão, doenças cardiovasculares, obesidade, síndrome metabólica e diabetes tipo 2, além de problemas ósseos como osteopenia e osteoporose (8).

O estresse não é apenas sobre o que acontece com uma pessoa, mas sobre como essa pessoa percebe e reage ao que acontece, sendo influenciado por fatores pessoais e contextuais (9). Um estudo brasileiro observou-se que fatores socioeconômicos, como idade entre 18 e 20 anos e ser do sexo feminino, estão associados ao estresse severo/extremo (4). A exposição a condições socioeconômicas adversas contribui para o estresse crônico, devido a ciclos de retorno que perpetuam a dificuldade de enfrentamento e a percepção de estressores como incontroláveis. Portanto, é essencial abordar o contexto socioeconômico para a eficácia das intervenções psicológicas (10-11).

Desse modo, alguns fatores clínicos, como histórico de ansiedade, depressão e uso de medicamentos também estão associados ao estresse (4). Um estudo canadense demonstrou que indivíduos com multimorbidade cardiometabólica têm maior probabilidade de relatar altos níveis de estresse, sendo maior entre aqueles com três condições cardiometabólicas. Ainda, há uma associação entre multimorbidade e comportamentos de estilo de vida, como atividade física e níveis de estresse, com esses indivíduos apresentando comportamentos de estilo de vida associados a desfechos negativos de saúde (12).

Diante do exposto, este estudo teve por objetivo verificar a percepção de estresse entre as residentes em Vitória, Espírito Santo, e sua associação com características socioeconômicas, comportamentais e clínicas.

## Métodos

### Delineamento

Trata-se de um estudo transversal, que faz parte de um estudo maior intitulado “Violência contra mulher: um estudo de base populacional em Vitória, Espírito Santo”.

### Contexto

O estudo foi realizado em Vitória, Espírito Santo, município que possui uma população de 327.801 habitantes, sendo 53,04% mulheres (13).

### Participantes

A amostra do estudo foi composta por mulheres com idade de 18 anos ou mais residentes em Vitória, que tinham ou tiveram parceiro íntimo nos últimos 24 meses.

Para definição da amostra, foram selecionados os setores censitários do Instituto Brasileiro de Geografia e Estatística de 2010. A seleção envolveu a divisão do número total de domicílios (108.515) por 100, resultando em um pulo sistemático de 1.085. O setor inicial foi escolhido aleatoriamente, e os outros 99 setores subsequentes foram escolhidos com base no pulo sistemático. Os domicílios foram selecionados aleatoriamente a partir de uma lista do Instituto Brasileiro de Geografia e Estatística. No total, foram entrevistadas 1.086 mulheres, sendo selecionada uma em cada domicílio.

### Variáveis

As variáveis independentes analisadas foram compostas por dados socioeconômicas, como: faixa etária (anos) (18 a 24; 25 a 34; 35 a 44; 45 a 59; 60 ou mais), raça/cor da pele (branca e não branca), escolaridade (anos de estudo) (0 a 8 anos; igual ou acima de 8 anos), situação conjugal (com companheiro; sem companheiro), renda familiar por quartil (1º quartil – mais pobres; 2º quartil; 3º quartil; 4º quartil – mais ricos), casa própria (não; sim), religião (não; sim), trabalho remunerado (não; sim), número de moradores no domicílio (0 a 2 pessoas; 3 a 5 pessoas; 6 ou mais pessoas), recebe auxílio do governo (não; sim) e plano de saúde (não; sim). Os dados comportamentais são compostos por: prática de atividade física (não; sim), consumo de bebidas alcoólicas (não; sim) e tabagismo (não; sim). Por fim, os dados clínicos são: estado nutricional (eutrófica, baixo peso, sobrepeso, obesidade) e multimorbidade (não; sim).

### Fonte de dados e mensuração

O desfecho do presente estudo diz respeito aos níveis de estresse percebidos, avaliados por meio do instrumento intitulado Perceived Stress Scale, em sua versão curta (short form) de dez questões, que permite avaliar a percepção de estresse com relação às experiências de vida no último mês (14). A escala no estudo foi traduzida e validada no Brasil (15). O instrumento do estresse utilizado foi a versão curta de dez questões, com opções de resposta que variam de 0 a 4 (0=nunca; 1=quase nunca; 2=às vezes; 3=quase sempre e 4=sempre) para as questões com conotação negativa. Já as questões com conotação positiva têm pontuação invertida (0=4; 1=3; 2=2; 3=1 e 4=0). O total da escala é a soma das pontuações destas dez questões, sendo que o escore pode variar de 0 a 40 (15).

### Coleta de dados

As entrevistas foram realizadas no domicílio das mulheres, no período de janeiro a maio de 2022. Em dezembro de 2021, foi realizado um estudo piloto, sendo entrevistadas 34 mulheres, não incluídas na amostra final. Para a coleta de dados, foram utilizados tablets, gerenciados com auxílio de ferramenta de captura eletrônica de dados Research Eletronic Data Capture.

### Métodos estatísticos

As análises foram conduzidas mediante apresentação de frequência bruta, relativa e intervalos de confiança de 95% (IC95%). Foi utilizada como medida de tendência central a apresentação de média (µ) para as variáveis contínuas com seu desvio-padrão (DP). A normalidade dos dados contínuos foi averiguada a partir de visualização do histograma de Skewness/Kurtosis confirmando a distribuição normal e não rejeição da hipótese nula pelo teste de Shapiro-Francia, igual a 0.14. O coeficiente dos dados foi avaliado quanto ao Alpha Scale de Cronbach (α), apresentando boa consistência interna (0,80), com confirmatória por ômega de Mcdonalds (ω) de 0,80. Em seguida, os dados foram avaliados mediante análise bivariada, por testes t para média quando as variáveis independentes fossem dicotômicas, e análise de variância por ANOVA pelas médias, quando as variáveis independentes apresentavam três ou mais categorias; nessa etapa, considerou-se significativo o p-valor 0,05. Para verificar a associação proposta, análises multivariáveis por regressão linear simples e múltipla foram expressas por seus betas (ß). Para essa etapa, o nível de significância para entrar no modelo multivariável foi de 20% e 5% , para que permanecesse no modelo final. Todas as etapas descritas da análise foram realizadas no programa estatístico Stata versão 17.0. 

## Resultados

A média (µ) de percepção de estresse entre as mulheres residentes de Vitória foi de 16,4 e desvio-padrão (DP) de 7,5. As maiores médias (µ=2,0; DP 1,^2^) foram encontradas quando as mulheres foram questionadas sobre estarem nervosas ou estressadas (µ=2,0; DP 1,^2^), e da questão sobre se sentirem bravas por causa de coisas que estiveram fora de seu controle (µ=2,0; DP 1,^3^) (Tabela 1).

**Tabela 1:** Média, mediana e desvio-padrão (DP) de estresse percebido entre mulheres. Vitória, 2022 (n=1.086)

No último mês, com que frequência	D^a^	Média±DP	Mediana
Você ficou aborrecida por causa de algo que aconteceu inesperadamente?	-	1,7±1,3	2,00
Você sentiu que foi incapaz de controlar coisas importantes na sua vida?	-	1,5±1,3	1,00
Você esteve nervosa ou estressada?	-	2,0±1,2	2,00
Você esteve confiante em sua capacidade de lidar com seus problemas pessoais?	+	1,1±1,1	1,00
Você sentiu que as coisas aconteceram da maneira que você esperava?	+	1,9±1,2	2,00
Você achou que não conseguiria lidar com todas as coisas que tinha por fazer?	-	1,8±1,2	2,00
Você foi capaz de controlar irritações na sua vida?	+	1,4±1,2	1,00
Você sentiu que todos os aspectos de sua vida estavam sob controle?	+	1,7±1,2	2,00
Você esteve brava por causa de coisas que estiveram fora de seu controle?	-	2,0±1,3	2,00
Você sentiu que os problemas acumularam tanto que você não conseguiria resolvê-los?	-	1,3±1,3	1,00
**Pontuação total do PSS-10**		16,4±7,5	17,00

^a^ D: Domínios da escala, sendo negativo (-) as respostas com conotações negativas e positivo (+) as respostas com conotações positivas; ^b^PSS-10: Perceived Stress Scale-10.

A maior média de percepção de estresse foi encontrada entre as mulheres com 18 a 24 anos (µ=20,^3^), não brancas (µ=16,^8^), com escolaridade igual ou acima de 8 anos (µ=16,^7^), pertencentes ao primeiro quartil de renda (µ=17,^5^), sem companheiros (µ=17,^8^), não religiosas (µ=16,0), que vivem com seis ou mais pessoas no mesmo domicílio (µ=17,^6^), que não possuem plano de saúde (µ=17,0), não têm casa própria (µ=17,^5^), recebem auxílio do governo (µ=18,^1^), não fazem atividade física (µ=17,^2^), são tabagistas (µ=17,^2^) e fazem uso de bebidas alcoólicas (µ=17,0) (Tabela 2). 

**Tabela 2:** Frequência bruta (n), frequência relativa (%), média e desvio-padrão (DP) da percepção de estresse de acordo com as características socioeconômicas, comportamentais e clínicas das mulheres. Vitória, 2022 (n=1.086)

Variáveis	n^a^ (%^b^ )	Média±DP	p-valor
**Faixa etária** (anos)			<0,001
18-24	105 (9,6)	20,3±7,0	
25-34	192 (17,6)	18,1±6,9	
35-44	227 (20,9)	17,4±7,9	
45-59	300 (27,7)	15,4±7,4	
60 (ou mais)	262 (24,1)	13,9±6,9	
**Raça/cor da pele**			0,040
Não Branca	650 (40,2)	16,8±7,8	
Branca	436 (59,8)	15,8±7,3	
**Escolaridade** (**anos de estudo**)			0,023
<8	203 (18,7)	15,3±7,6	
≥8	883 (81,3)	16,7±7,5	
**Renda familiar** (quartil)			0,003
1º (mais pobres)	290 (26,7)	17,5±7,6	
2º	288 (26,5)	16,2±7,3	
3º	238 (21,9)	16,7±7,3	
4º (mais ricos)	270 (24,9)	15,2±7,6	
**Situação conjugal**			0,004
Com companheiro	886 (81,6)	16,1±7,5	
Sem companheiro	200 (18,4)	17,8±7,3	
**Religião**			<0,001
Não	182 (16,8)	18,5±8,3	
Sim	904 (83,2)	16,0±7,3	
**Trabalho remunerado**			0,531
Não	598 (55,1)	16,3±7,5	
Sim	488 (44,9)	16,7±7,8	
**Número de pessoas no domicílio**			0,004
0-2	412 (37,9)	15,5±7,5	
3-5	624 (57,5)	16,9±7,5	
≥6	50 (4,6)	17,6±6,2	
**Possui plano de Saúde**			0,011
Não	514 (47,3)	17,0±7,7	
Sim	572 (52,7)	15,9±7,7	
**Possui casa própria**			0,002
Não	317 (29,2)	17,5±7,9	
Sim	769 (70,8)	15,9±7,3	
**Auxílio do governo**			<0,001
Não	895 (82,4)	16,0±7,3	
Sim	191 (17,6)	18,1±8,2	
**Atividade Física**			<0,001
Não	589 (54,2)	17,2±7,6	
Sim	497 (45,8)	15,5±7,3	
**Tabagismo**			0,029
Não	804 (74,0)	16,1±7,5	
Sim	282 (26,0)	17,2±7,5	
**Uso de bebidas alcoólicas**			0,008
Não	548 (50,7)	15,8±7,45	
Sim	532 (49,3)	17,0±7,6	
**Estado nutricional**			0,078
Eutrofia	449 (41,4)	16,4±7,4	
Baixo peso	33 (3,0)	16,2±7,6	
Sobrepeso	356 (32,8)	15,8±7,6	
Obesidade	246 (22,8)	17,4±7,6	
**Multimorbidade**			0,196
Não	598 (55,1)	16,1±7,2	
Sim	488 (44,9)	16,7±7,8	

Observa-se, na Tabela 3, na análise bruta, que a percepção do estresse esteve relacionada à idade, escolaridade, renda familiar, situação conjugal, religião, número de pessoas em domicílio, se tem plano de saúde, casa própria e recebe auxílio do governo, assim como a prática de atividade física, o tabagismo e o uso de bebidas alcoólicas (p-valor<0,05) (Tabela 3).

**Tabela 3:** Coeficiente de regressão linear (β) simples e intervalos de confiança de 95% (IC95%) da associação entre a percepção de estresse e as características socioeconômicas, comportamentais e clínicas entre mulheres. Vitória, 2022 (n=1.086)

Variáveis	β (IC95%)	p-valor
**Faixa etária** (anos)		<0,001
18-24 anos	1,0	
25-34 anos	-2,20 (-3,93; -0,47)	
35-44 anos	-2,96 (-4,64; -1,28)	
45-59 anos	-4,90 (-6,51; -3,28)	
60 (ou mais)	-6,42 (-8,07; -4,78)	
**Raça/cor da pele**		0,040
Não Branca	0,95 (0,43; 1,86)	
Branca	1,0	
**Escolaridade** (anos)		0,023
<7	1,33(0,19; 2,48)	
≥8	1,0	0,003
**Renda familiar** (quartil)		
1º (mais pobres)	2,28 (1,04; 3,52)	
2º	0,96 (-0,29; 2,20)	
3º	1,52 (0,22; 2,82)	
4º (mais ricos)	1,0	
**Situação conjugal**		0,004
Com companheiro	1,0	
Sem companheiro	1,68 (0,53; 2,82)	
Religião		<0,001
Não	2,55 (1,36; 3,74)	
Sim	1,0	
**Trabalho remunerado**		0,530
Não	-2,87 (-1,19; 0,61)	
Sim	1,0	
**Número de pessoas no domicílio**		0,004
0-2	1,0	
3-5	1,47 (0,54; 2,40)	
6 (ou mais pessoas)	2,14 (-0,55; 4,34)	
**Possui plano de saúde**		0,011
Não	1,16 (0,26; 2,05)	
Sim	1,0	
**Possui casa própria**		0,002
Não	1,54 (0,56; 2,52)	
Sim	1,0	
**Auxílio do governo**		<0,001
Não	1,0	
Sim	2,08 (0,91; 3,25)	
**Atividade física**		<0,001
Não	1,65 (0,76; 2,54)	
Sim	1,0	
Tabagismo		0,029
Não	1,0	
Sim	1,31 (0,11; 2,15)	
**Uso de álcool** (n=1.080)		0,008
Não	1,0	
Sim	1,22 (0,32; -2,11)	
**Estado nutricional** (n=1.084)		0,078
Eutrofia	1,0	
Baixo peso	-1,07 (-2,76; 2,55)	
Sobrepeso	-0,55 (-1,60; 0,49)	
Obesidade	1,06 (-0,10; 2,23)	
Multimorbidade		0,196
Não	1,0	
Sim	0,59 (-0,30; 1,49)	

Na análise ajustada, apresentada na Tabela 4, mulheres com 60 anos ou mais apresentaram, em média, 6,04 pontos a menos de percepção de estresse, em comparação à faixa mais jovem (18 a 24 anos). O grupo pertencente ao primeiro quartil de renda familiar, ou seja, os mais pobres, apresentaram, em média, 1,35 ponto a mais de estresse, quando comparadas às mais ricas, e aquelas que recebem auxílio do governo apresentam 1,86 ponto a mais de estresse, comparadas às que não recebem auxílio. Mulheres sem religião tiveram, em média, 1,21 ponto a mais de estresse percebido comparadas às mulheres que possuem religião. Maiores médias de estresse foram encontradas entre as mulheres que não praticavam atividade física (β=1,46), se declararam tabagistas (β=1,^22^) e tinham alguma multimorbidade (β=2,^38^) (p-valor<0,050) (Tabela 4).

**Tabela 4:** Coeficiente de regressão linear (β) múltipla e intervalos de confiança de 95% (IC95%) da associação entre a percepção de estresse e as características socioeconômicas, comportamentais e clínicas entre mulheres. Vitória, 2022 (n=1.086)

Variáveis	β ajustado (IC95%)	p-valor
**Faixa etária** (anos)		<0,001
18-24	1,0	
25-34	-2,23 (-3,95; -0,51)	
35-44	-2,79 (-4,46; -1,11)	
45-59	-4,63 (-6,26; -3,00)	
60 (ou mais)	-6,04 (-7,71; -4,38)	
**Raça/cor da pele**		0,799
Não Branca	0,12 (-0,83; -1,07)	
Branca	1,0	
**Escolaridade** (anos)		0,280
<7	0,67 (-0,55; -1,89)	
≥8	1,0	
**Renda familiar** (quartil)		0,003
1º (mais pobres)	1,35 (0,92; -2,61)	
2º	0,96 (-0,25; -2,17)	
3º	1,58 (0,32; -2,85)	
4º (mais ricos)	1,0	
**Situação conjugal**		0,142
Com companheiro	-0,85 (-1,98; -0,28)	
Sem companheiro	1,0	
Religião		0,046
Não	1,21 (0,19; -2,40)	
Sim	1,0	
**Número de pessoas no domicílio**		0,110
0-2	1,0	
3-5	1,02 (0,07; -1,97)	
6 (ou mais pessoas)	0,48 (-1,70; -2,65)	
**Possui plano de saúde**		0,805
Não	-0,14 (-1,23; -0,95)	
Sim	1,0	
**Possui casa própria**		0,770
Não	0,15 (-0,86; -1,16 )	
Sim	1,0	
**Auxílio do governo**		0,001
Não	1,0	
Sim	1,86 (0,73; -2,99)	
**Atividade física**		0,001
Não	1,46 (0,60; -2,32)	
Sim	1,0	
Tabagismo		0,015
Não	1,0	
Sim	1,22 (0,23; -2,19)	
**Uso de álcool** (n=1.080)		0,935
Não	1,0	
Sim	0,04 (-0,86; -0,95)	
**Estado nutricional** (n=1.084)		0,407
Eutrofia	1,0	
Baixo peso	-1,23 (-3,75; -1,30)	
Sobrepeso	-0,06 (-1,06; -0,95)	
Obesidade	0,67 (-0,46; -1,81)	
Multimorbidade		<0,001
Não	1,0	
Sim	2,38 (1,44; -3,32)	

## Discussão

Neste estudo, foi possível identificar a média de percepção de estresse entre as mulheres residentes de Vitória (µ=16,4; DP 7,51). Observam-se maiores médias de estresse entre as mulheres mais jovens (18 a 24 anos), pertencentes ao grupo mais pobre, que recebem auxílio do governo, e declaram não ter religião. Além disso, não praticar atividade física, ser tabagista e apresentar alguma multimorbidade estiveram associados ao aumento do estresse entre as participantes.

A idade apresentou associação negativa com os níveis de estresse, com mulheres mais jovens (18-24 anos) reportando os níveis mais altos. Um estudo de base populacional, realizado em um município no sul do Brasil, revelou que mulheres, especialmente na faixa etária de 18 a 39 anos, constituíam um dos grupos mais estressados (16). Este fenômeno pode ser atribuído ao acúmulo de responsabilidades em múltiplos papéis sociais, familiares e profissionais, além de demandas pessoais, biológicas e sociais, contribuindo significativamente para o aumento do estresse (17). De maneira semelhante, um estudo com idosos de São Paulo e Paraíba evidenciou que eles se consideravam, de forma geral, mais satisfeitos com a vida em comparação às pessoas mais jovens, sugerindo que, segundo a teoria da seletividade socioemocional, as emoções são mais bem reguladas com o envelhecimento, resultando em maior sentimento de bem-estar (18).

Corroborando nossos resultados em relação à renda familiar, um estudo dinamarquês que investigou as associações entre estresse percebido, status socioeconômico e comportamento de risco à saúde encontrou *odds ratios* (OR) ajustados de estresse percebido maiores entre moradores com baixa renda mensal (OR 1,57; IC95% 1,35; 1,83), em comparação com aqueles com renda mensal elevada (19). Esse achado pode ser justificado pelo conceito de tensão financeira, definido como um tipo específico de estresse causado por dificuldades econômicas, como instabilidade financeira, acúmulo de dívidas e histórico de crédito prejudicado (20). Embora a pobreza esteja diretamente relacionada à renda, a tensão financeira é uma experiência emocional subjetiva, sem ligação com um valor de renda fixo (21).

Tanto a pobreza quanto a tensão financeira afetam negativamente a saúde mental, conforme demonstram estudos anteriores (22-23). No caso das mães, a tensão financeira está associada a aumento nos sintomas de depressão (24). Em famílias com filhos pequenos, essa pressão econômica pode levar a decisões difíceis, comprometendo a qualidade dos cuidados parentais e aumentando os sentimentos de culpa, o que agrava ainda mais os problemas de saúde mental (25).

Outro fator relevante é o apoio governamental, visto que mulheres que declararam receber auxílio do governo relataram níveis mais elevados de estresse em comparação com aquelas que não recebem esse auxílio. Sendo assim, um estudo com 433 participantes cadastrados no Cadastro Único do Governo Federal em Belém, incluindo mães ou responsáveis por filhos de 5 a 18 anos, indicou que 96,5% dos cuidadores principais eram mulheres. Esse estudo mostrou que as famílias mais pobres apresentavam níveis mais altos de estresse parental, ou seja, o estresse associado às responsabilidades e exigências do cuidado com os filhos. Esses dados reforçam que as famílias elegíveis ao auxílio governamental eram economicamente mais vulneráveis, corroborando a discussão prévia sobre os impactos da pobreza na saúde mental (26).

Além da renda e do apoio social, as participantes sem religião apresentaram maior percepção de estresse em comparação com aquelas com crenças religiosas. Um estudo com 795 pessoas investigou como as conexões espirituais auxiliaram no enfrentamento do estresse e da ansiedade nas primeiras fases da pandemia de covid-19. Mulheres e jovens são mais vulneráveis ao estresse e à ansiedade; no entanto, conexões espirituais podem atuar como recurso protetor, especialmente em tempos de crise, reduzindo a probabilidade de sintomas de estresse e ansiedade. A maior suscetibilidade das mulheres ao estresse pode estar ligada à reatividade elevada e ao uso menos eficaz de estratégias de enfrentamento (27). Embora a conexão espiritual ofereça conforto, pode servir mais como alívio temporário do que como solução duradoura para esse grupo (28).

Dessa forma, a espiritualidade e a religiosidade podem ser agentes de alívio em momentos de estresse, atuando como estratégias de adaptação que aumentam a resiliência ao longo da vida e contribuem para a manutenção da qualidade de vida e da saúde (29).

No que diz respeito à atividade física, mulheres que não praticam atividades físicas apresentaram percepção de estresse mais elevada do que aquelas que se exercitam regularmente. Um estudo realizado em Aracaju, com uma amostra predominantemente feminina (51%), revelou que a falta de atividade física está associada a níveis mais altos de estresse (30). A atividade física regular está associada a melhorias na autoestima, no autoconceito, na percepção da imagem corporal, nas funções cognitivas e nas relações sociais, além de contribuir para a redução de estresse e ansiedade. Incorporar hábitos saudáveis é fundamental para promover a saúde mental. Assim, a atividade física emerge como uma ferramenta crucial para o bem-estar (31).

O tabagismo está associado à maior percepção de estresse. Mulheres que fumam ou já fumaram relataram, em média, 1,23 ponto a mais de estresse em comparação às não fumantes, reafirmando estudos anteriores (32-33). Fumar é frequentemente usado como um mecanismo de enfrentamento ao estresse, proporcionando uma pausa em ambientes adversos, como moradias de baixa qualidade, vizinhanças desfavoráveis ou em situações de trânsito (32). Essa prática é vista como uma estratégia para aliviar questões cotidianas (33).

Em relação às condições de saúde, mulheres com diagnóstico de multimorbidade relataram maior percepção de estresse em comparação àquelas sem diagnósticos múltiplos, o que pode ser justificado pelo sofrimento relacionado às doenças e aos estressores diários enfrentados por pessoas com condições crônicas (34-35). Em um estudo transversal de base populacional, realizado com adultos mais velhos em países de baixa e média renda, destacou-se que condições crônicas e multimorbidade estavam associadas a níveis mais altos de estresse. Ademais, o estresse percebido, aliado às condições crônicas, mostrou-se relacionado a piores resultados de saúde, evidenciando a necessidade urgente de intervenções integradas e de baixo custo em nível populacional para lidar com o estresse em indivíduos com doenças crônicas (36).

Esses achados destacam a complexidade do estresse. Estudos que exploram a percepção desse agravo de saúde em relação às características socioeconômicas, comportamentais e clínicas oferecem uma compreensão mais detalhada das causas e impactos desse fenômeno (26,37). O estresse, “um vilão invisível”, exige políticas públicas que enfrentem desigualdades sociais e promovam estratégias eficazes de gerenciamento para mitigar seus efeitos. Medidas como a promoção da igualdade de gênero, o acesso à educação, emprego, alimentos saudáveis, lazer e atividades físicas são fundamentais. Além disso, o rastreamento do estresse pelos profissionais de saúde é essencial para identificar comorbidades e viabilizar intervenções precoces. A inclusão de psicólogos nas equipes de saúde da família pode ampliar ainda mais o acesso a cuidados em saúde mental (38).

O estudo possui limitações que precisam ser consideradas, como o delineamento transversal que impossibilita inferir causalidade, permitindo apenas projetar possíveis associações entre os dados avaliados, entretanto, advém de uma pesquisa de base populacional representativa da população, fortalecendo as relações examinadas. Outra limitação é a possibilidade de viés de informação, o que pode influenciar na aferição da percepção de estresse, todavia; a metodologia de coleta de dados utiliza um instrumento validado para rastreio e se assemelha à de outras pesquisas com o tema, e os achados do presente estudo vão ao encontro da literatura.

Por fim, este estudo permite concluir que o perfil socioeconômico, comportamental e clínico das mulheres se apresenta associado ao fenômeno do estresse. Desse modo, é importante orientar o desenvolvimento de estratégias de intervenção e políticas públicas mais eficazes, que abordem não somente os aspectos físicos, mas também psicológicos do estresse, considerando os diferentes contextos culturais e suas demandas específicas. Nesse sentido, essa perspectiva holística da mulher tem o potencial de transformar a abordagem a esse grupo, integrando-o a um contexto mais amplo e promovendo avanços capazes de impactar positivamente as vidas das mulheres. É importante ponderar que, embora desafiadora, essa proposta também representa uma oportunidade única para liderar mudanças significativas e sustentáveis no cuidado à saúde.

## Data Availability

O banco de dados e os códigos de análise utilizados na pesquisa estão disponíveis em https://doi.org/10.48331/scielodata.C8WFB1.
